# Integrated analysis of mutations, miRNA and mRNA expression in glioblastoma

**DOI:** 10.1186/1752-0509-4-163

**Published:** 2010-11-29

**Authors:** Hua Dong, Li Luo, Shengjun Hong, Hoicheong Siu, Yanghua Xiao, Li Jin, Rui Chen, Momiao Xiong

**Affiliations:** 1 State Key Laboratory of Genetic Engineering and MOE Key Laboratory of Contemporary Anthropology, School of Life Sciences and Institutes of Biomedical Sciences, Fudan University, Shanghai, 200433, China; 2 Human Genetics Center, The University of Texas School of Public Health, Houston, TX 77030, USA; 3 Department of Computing and Information Technology, Shanghai (International) Database Research Center, Fudan University, Shanghai, 200433, China; 4 Department of Molecular and Human Genetics, Baylor College of Medicine, Houston, TX 77030, USA

## Abstract

**Background:**

Glioblastoma arises from complex interactions between a variety of genetic alterations and environmental perturbations. Little attention has been paid to understanding how genetic variations, altered gene expression and microRNA (miRNA) expression are integrated into networks which act together to alter regulation and finally lead to the emergence of complex phenotypes and glioblastoma.

**Results:**

We identified association of somatic mutations in 14 genes with glioblastoma, of which 8 genes are newly identified, and association of loss of heterozygosity (LOH) is identified in 11 genes with glioblastoma, of which 9 genes are newly discovered. By gene coexpression network analysis, we indentified 15 genes essential to the function of the network, most of which are cancer related genes. We also constructed miRNA coexpression networks and found 19 important miRNAs of which 3 were significantly related to glioblastoma patients' survival. We identified 3,953 predicted miRNA-mRNA pairs, of which 14 were previously verified by experiments in other groups. Using pathway enrichment analysis we also found that the genes in the target network of the top 19 important miRNAs were mainly involved in cancer related signaling pathways, synaptic transmission and nervous systems processes. Finally, we developed new methods to decipher the pathway connecting mutations, expression information and glioblastoma. We indentified 4 cis-expression quantitative trait locus (eQTL): TP53, EGFR, NF1 and PIK3C2G; 262 trans eQTL and 26 trans miRNA eQTL for somatic mutation; 2 cis-eQTL: NRAP and EGFR; 409 trans- eQTL and 27 trans- miRNA eQTL for lost of heterozygosity (LOH) mutation.

**Conclusions:**

Our results demonstrate that integrated analysis of multi-dimensional data has the potential to unravel the mechanism of tumor initiation and progression.

## Background

Glioblastoma (glioblastoma multiforme or GBM) is the most common and aggressive type of primary brain tumor in humans, involving glial cells and accounting for 52% of all parenchymal brain tumor cases and 20% of all intracranial tumors [1]. Glioblastoma is located preferentially in the cerebral hemispheres. In the past two decades, the molecular mechanisms, genetics and pathways to treat GBM have extensively been studied [2]. However, the precise mechanism of GBM is unknown and its median survival rate is very low [3]. The Cancer Genome Atlas (TCGA) generated large-scale multi-dimensional datasets to catalogue cancer alterations [4]. GBM is the first cancer studied by TCGA. In the GBM pilot project, a total of 601 genes were sequenced for detection of somatic mutations in 179 tumor and matched normal tissues pairs; expressions of 12,042 genes were measured in 243 tumor tissue samples and 10 normal tissue samples and 1 cell line; and expressions of 534 microRNAs (miRNAs) were profiled in 240 tumor tissue samples and 10 normal tissue samples. The generated multi-dimensional genetic and molecular data will provide rich information which allows us to uncover mechanisms of GBM.

**Figure 1:**
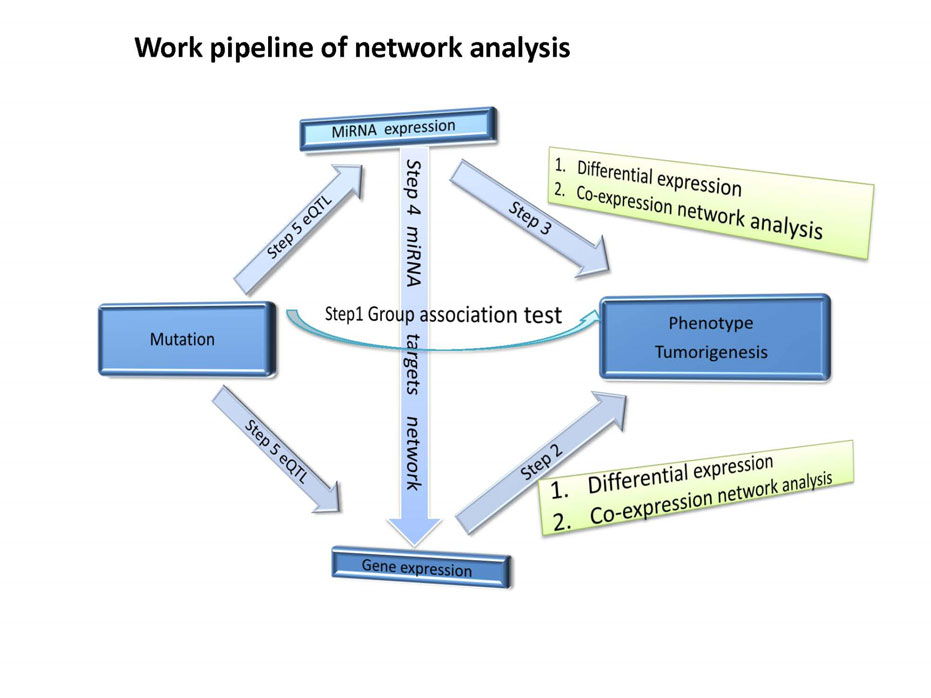
**The pipeline of the GBM network analysis approach**.

In the past, although enormous efforts toward generating high-throughput genetic and molecular data have been made, knowing how to integrate genetic and molecular data and unravel underlying mechanisms of cancer remains unresolved. To gain significant insights into the mechanisms of cancer, it is indispensable to reconstruct and dissect various pathways, and genetic and molecular networks that define the molecular states of systems involved in tumorigenesis. To meet this challenge, we will reconstruct genetic and molecular networks involved in tumorigenesis and study how these networks respond to the perturbation of somatic mutations and environments. Genes with DNA variation, mRNA and miRNA form the complex genetic and molecular networks that determine the cell's function and response to the perturbation of external stimuli. These networks consist of three levels. The subnetwork in the first level consists of (1) significantly associated somatic mutations and (2) LOH which were identified by a group association test statistic. The subnetworks in the third level are mRNA and miRNA coexpression networks. The subnetworks in the second level are to connect the subnetworks in the first level and subnetworks in the third level. The network pipeline analysis approach is shown in Figure 1.

It is increasingly recognized that miRNAs have emerged as an important component in the regulation of gene expression, with imperfect base pairing, to target sites in the 3' UTR of the messenger RNAs [5]. Three types of methods, sequence analysis, miRNA-mRNA regression analysis, and machine learning, have been used to identify potential target genes [6,7]. To improve the accuracy of target prediction, we will combine sequence analysis with regression analysis for target prediction, which finally leads to the formation of miRNA target networks. miRNA target networks connect gene coexpression and miRNA coexpression networks. Expression quantitative trait loci (eQTLs) are genomic loci that regulate gene transcription. eQTLs may act in cis (locally) or trans (at a distance) to a gene or a precursor miRNA [8]. We applied a group regression method which regresses the expression of a mRNA or a miRNA on the number of all mutated alleles across the region of interest to identify eQTL. The identified eQTLs connected the subnetworks in the first and third levels.

Biological functions and mechanisms are encoded in network properties. An important strategy for unraveling the mechanisms of initiation and progression of cancer is to conduct analysis of complex genetic and molecular networks and study their behaviors under genetic and environment perturbations. Robustness of a biological network, ability to retain much of its functionality in the face of perturbation [9], has emerged as a fundamental concept in the study of network topological properties [10]. The locations of the DNA variants, mRNA and miRNA in the genetic and molecular networks are likely to affect the phenotypes. We use network structural analysis as a tool to identify a set of key cancer causing genome alternations and core modules of biological networks that play an essential role in the development of cancer.

The purpose of this report is to use systems biology and network approaches to develop novel analytical strategies to unravel the mechanism of GBM by systematically integrating the multi-dimensional datasets from the TCGA project.

## Results

### Test Association of Somatic Mutations and LOH Mutations with Glioblastoma

Traditional statistical methods for genetic studies of complex diseases are mainly based on the common disease/common variant hypothesis. However, it has been reported that common variants account for only a small proportion of the genetic contribution to complex diseases. Recent deep-resequencing reveals that in the genome there are a large number of rare variants (0.05 × *MAF*) which also play an important role in causing various complex diseases including cancer [11]. Multiple rare mutations, each with a minor marginal genetic effect, but collectively may make large contributions in the population. Most statistics for testing the association of common alleles with common diseases have mainly focused on the investigation of variants individually. However, due to their rarity, the frequencies of rare alleles may be comparable with genotyping errors. As a consequence, individual tests of association of rare variants with disease, as is often done by the traditional association tests, have limited power and may not be robust [12]. An alternative approach to the current variant-by-variant tests is group association tests in which a group of rare genetic variants are jointly tested [12-14]. It has been shown that the number of rare alleles in large samples is approximately distributed as a Poisson process with its intensity depending on the total mutation rate [15]. The intensity of the Poisson process within a segment of the genome can be interpreted as the mutation rate. Similar to the standard χ^2 ^test for association of SNPs which compares the difference in allele frequencies between cases and controls, we propose a new group test statistic *T_a _*that is to compare differences in the mutation rates between tumor and normal samples (for details, see Methods). The statistic *T_a _*was applied to GBM in the TCGA dataset. The tumor tissues of 179 GBM patients and 179 matched normal tissues were sequenced. A somatic mutation was recorded when the mutation was detected only in the tumor tissue. There are 306 genes in which at least one somatic mutation was detected. A LOH mutation was recorded when the genotype in blood or normal tissue is heterozygous, and in the tumor tissue, the reference allele loses normal function and the genotype becomes homozygous. There are 124 genes in which at least one LOH mutation was detected. We identified association of somatic mutations in 14 genes by the statistics *T_a _*(Table 1), and association of LOH in 11 genes by the statistic *T_G _*with GBM with a false discovery rate (FDR) of less than 0.05 (Table 2). Genes *TP53, PTEN, EGFR, NF1, RB1 *and *ERBB2 *were reported to be associated with GBM in the previous TCGA data analysis [4]. The remaining 8 somatic mutated genes and 10 LOH mutated genes were newly identified by the statistics *T_G _*or *T_a_*. NCBI Entrez gene database [16] reported that: *CHEK2 *is a cell cycle checkpoint regulator and putative tumor suppressor, and associated with GBM; *GSTM5 *was reported to be involved in cancer, *BCL11A *is a proto-oncogene, and *FN1 *is involved in tumor metastasis and angiogenesis. Gene *PRAME *was reported to be associated with melanoma and acute leukemias. Association of the other 9 genes such as *NRAP, MK167, C10orf54 *and *C9orf66 *with GBM was first reported here.

**Table 1:** P-values for testing association of somatic mutations with the GBM.

Gene	p-value	FDR	Mutation frequency
TP53	3.46E-11	5.90E-10	0.1453
PTEN	2.25E-07	1.92E-06	0.0698
EGFR	1.22E-06	6.92E-06	0.0587
FKBP9	1.38E-04	5.89E-04	0.0363
CHEK2	1.39E-03	4.73E-03	0.0475
GSTM5	2.41E-03	6.86E-03	0.0251
DST	4.28E-03	8.12E-03	0.0223
RB1	3.54E-03	8.62E-03	0.0251
NF1	4.17E-03	8.89E-03	0.0363
BCL11A	1.36E-02	2.33E-02	0.0168
ERBB2	1.57E-02	2.43E-02	0.0307
PIK3C2G	2.45E-02	3.49E-02	0.0140
FN1	3.30E-02	4.33E-02	0.0168
COL3A1	4.46E-02	4.75E-02	0.0112

**Table 2:** P-values for testing association of LOH.

Gene	P-value	FDR	Mutation frequency
NRAP	5.71E-07	6.24E-06	0.1173
MKI67	8.19E-07	4.47E-06	0.1229
C10orf54	1.02E-04	3.70E-04	0.0391
C9orf66	1.63E-04	4.46E-04	0.0419
MYO3A	6.40E-04	1.40E-03	0.0307
PRAME	2.14E-03	3.90E-03	0.0251
EGFR	3.20E-03	4.99E-03	0.0279
IL1RL1	7.11E-03	9.71E-03	0.0196
HLA-DOA	1.30E-02	1.57E-02	0.0168
ABCA13	2.37E-02	2.59E-02	0.0140
CYP1B1	2.37E-02	2.59E-02	0.0140

### Network Analysis of Gene Expressions

Comparative studies of gene expression between normal and tumor tissues is one of the most widely used strategies for unraveling the molecular circuitry underlying cancer [17]. To uncover the mechanisms of glioblastoma, expressions of 12,042 genes were measured in 243 tumor tissue samples and 10 normal tissue samples and 1 cell line using the Affymetrix HT Human Genome U133 Array Plate Set. A total of 1,697 genes were differentially expressed between tumor and normal tissues by the Wilcoxon rank-sum test (P-value for declaring significance after Bonferroni correction is 4.15 × 10^-6^). Of the 1,697 genes, 97 genes were cancer genes or cancer candidate genes (Additional files 1); 11 of them were oncogene including *CDK4 *and *RAF4*; and 21 of them were tumor suppressor genes including *TP53 *and *RB1*. Twenty-five of which were GBM related genes including *TCF12, TP53, COL4A1, COL3A1 *and *COL5A2*. We also observed that of the 1,697 genes, 242 genes were in signal transduction pathways, 908 genes were down regulated and 789 genes were up regulated (Additional files 2).

**Figure 2:**
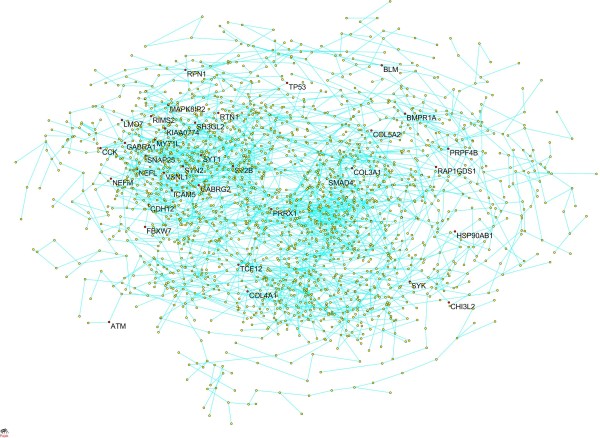
**The largest connected gene coexpression network**. The network had 2,115 genes and 2,276 edges. Genes related to GBM were highlighted in red.

**Figure 3:**
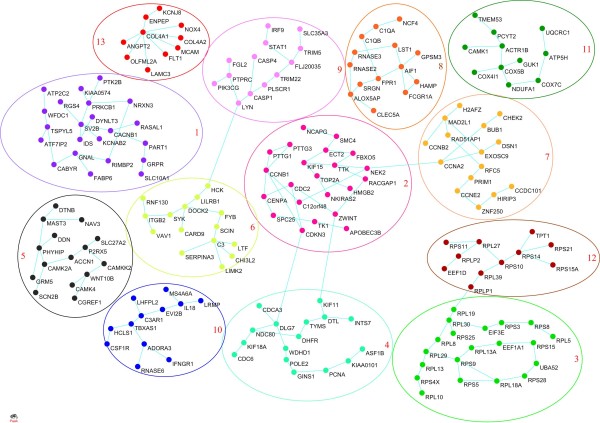
**13 functional modules in the largest connected gene coexpression network**. The modules were indexed by the number and also represented by color. Enriched pathways in the modules were listed in Table 3.

To investigate the functions of the genes at the system-level and uncover the mechanism of GBM, we used Lasso algorithms for a Gaussian graphical model to infer gene coexpression networks (see Methods). The largest connected coexpression network with the average shortest path 20.4 and diameter 74 had 2,115 genes and 2,276 edges (Figure 2). Coexpression networks are usually organized into modules that perform specific biological processes and tasks. A dynamic tree cut procedure was used to identify modules and a Fisher's exact test was used to discover pathways that were overrepresented in the module (see Methods). A total of 13 modules were significantly enriched for at least one pathway (Figure 3 and Table 3), indicating that a coexpression network was organized into functional units. Enriched pathways in the modules were involved in neurodegenerative diseases, development processes, cancer related signaling pathways and metabolism. A Parkinson's disease pathway was enriched in module 11 with olive green color. Genes *UQCRC1, ATP5 H, COX7C, NDUFA1, COX5R *and *COX4I1 *in module 11 are involved in mitochondria dysfunction http://www.genome.jp/kegg/pathway/hsa/hsa05012.html. A cell cycle pathway was enriched in module 7 with yellow color. We can see that coexpressed genes *CHEK2, CCNB2, CCNA2, CCNE2, MAD2L1 *and *BUB1 *in module 7 are directly or indirectly connected in the cell cycle pathway http://www.genome.jp/dbget-bin/show_pathway?hsa04110.

**Table 3:** 13 Modules in gene coexpression network in Figure 2 with their enriched pathways.

Module	Module color	Pathway category	Name	P-value
1	Purple	Cell signaling	Ion channel and phorbal esters signaling	8.60E-03

2	Wind stawberry	Apoptosis	Apoptotic DNA fragmentation and tissue homeostasis	3.52E-02
		Cell cycle regulation	Activation of Src by protein-tyrosine phosphatase alpha	4.23E-02
		
		Cell cycle regulation	AKAP95 role in mitosis and chromosome dynamics	4.98E-02
		
		Cell cycle regulation	Sonic Hedgehog (SHH) Receptor Ptc1 regulates cell cycle	4.23E-02

3	Forest green	Translation	Ribosome	3.32E-09

4	Teal blue	Metabolism of cofactors and vitamins	One carbon pool by folate	4.72E-02

5	Black	Cell signaling	Ca++/Calmodulin-dependent protein kinase Activation	3.59E-03

6	Green yellow	Immunology	Cells and molecules involved in local acute inflammatory response	4.68E-02

7	Yellow	Cell growth and death	Cell cycle	2.62E-02

8	Orange	Immunology	Classical complement	2.85E-02

9	Lavender	Cell signaling	IFN alpha signaling	1.03E-02

10	Blue	Cell activation	Th1/Th2 differentiation	3.79E-02

11	Olive green	Metabolism	Electron transport reaction in mitochondria	1.31E-02
		
		Energy metabolism	Oxidative phosphorylation	9.00E-03
		
		Neuro-degenerative diseases	Parkinson's disease	8.67E-03

12	Maroon	Translation	Ribosome	1.13E-03

13	Red	Metabolism/Neuroscience	Vitamin C in the brain	9.11E-03
		
		Cell activation	Angiotensin-converting enzyme 2 regulates heart function	1.27E-02
		
		Metabolism	Intrinsic prothrombin activation	3.83E-02
		
		Metabolism	Platelet amyloid precursor protein	1.47E-02
		
		Hematopoiesis	Regulators of bone mineralization	2.58E-03

The architecture of a coexpression network is important for uncovering the genes which are involved in cancer. To identify the most important genes in the coexpression network, we used the damage value of a node as a measure to rank the importance of a node. The damage value of a node which is defined as the difference in the number of nodes of the largest connected network before and after removal of that particular node can be used to measure the effect of the removal of the node, i.e. the ability of a network to avoid malfunctioning when a gene is removed (damaged) [18]. We ranked all differentially expressed genes in the largest coexpression subnetwork according to their damage values. The top 5% of the genes in the ranked list with damage values greater than 15 were summarized in Additional files 3. These genes were essential to the function of coexpression networks. We suspect that these genes may be involved in the development of GBM.

There were three differentially expressed genes (*TP53, COL3A1*, and *RAP1GDS1*) whose damage values ranked among the top in cancer and cancer candidate genes (Additional files 1). Their removal from the network may disconnect some components in the network and hence compromise the functions of the genes in coexpression networks. *TP53 *and *COL3A1 *are GBM related genes and *RAP1GDS1 *is a cancer candidate gene. The p53 pathway is central to oncogenesis [19]. *TP53 *was up regulated in GBM tissue samples and was highly over expressed in the CEREBRUM tissue samples. Interestingly it was observed that *TP53 *had a damage value of 23, but was only connected with two up regulated genes (*TGIF2 *and *EIF4A1*). *TGIF2 *was differentially expressed (P-value < 1.43 × 10^-7^) and had a damage value of 24. TGIF2 plays an oncogenic role through inhibition of *TGFB *[20]. *EIF4A1 *was over expressed in tumor tissue (P-value < 5.09 × 10^-6^) and had a damage value of 22. Using Programmed Cell Death 4 (*PDCD4*) *EIF4A1 *inhibits translation initiation and acts as a tumor suppressor by forming a complex [21]. *COL3A1 *was over expressed in GBM tissue samples (P-value < 1.6 × 10^-6 ^) and had damage value 21. It was also reported to be over expressed in ovarian cancer and breast cancer [22,23]. *COL3A1 *encodes a fibrillar collagen which is a major component of the extracellular matrix protein surrounding cancer cells. The presence of ECM protein prevents apoptosis of cancer cells. *COL3A1 *plays an important role in apoptosis, proliferation regulation and anticancer drug resistance [24]. *RAP1GDS1 *was under expressed in GBM tissue samples (P-value < 4.4 × 10^-7^) and had a damage value of 18 in the coexpression network. *RAP1GDS1 *is a transcription factor [25]. It was reported that translocation fusion of the *NUP98 *and *RAP1GDS1 *genes was recurrent in T-cell acute lymphocytic leukemia [26].

Other genes at the top of the list including *T1A1, KIAA1279 *and *CACYBP *also have remarkable biological implications. T1A1t had the largest damage value (38), was over expressed in the GBM tissue samples, and played a role in apoptosis [27]. *KIAA1279 *was reported to be associated with the nervous system [28] and *CACYBP *was reported to participate in p53-induced beta-catenin degradation. *CACYBP *can suppress proliferation and tumorigenesis of renal cancer cells [29]. *CACYBP *is also reported to be under expressed in gastric cancer [30] and renal cancer [29].

Using DAVID Bioinformatics Resources online [31], we performed the gene set enrichment analysis and found the most enriched Gene Ontology (GO) and pathway groups. GO:0045202~synapse (a cellular component term), GO:0007268~synaptic transmission (a biological process term) and GO:0005509~calcium ion binding (a molecular function term) were among the most significant GO terms, with P-values 6.98 × 10-^14^, 1.43 × 10^-9 ^and 5.06 × 10^-6^, respectively. Epithelial cell signaling in Helicobacter pylori infection (an infectious diseases pathway), long-term potentiation (a nervous system pathway) and calcium signaling pathway (a signal transduction pathway), were the most significantly enriched pathways with P-values 2.57 × 10^-6^, 4.42 × 10^-5 ^and 4.45 × 10^-5^, respectively. The results suggested that the differentially expressed genes were most involved in the signal and nervous system related pathways.

### Network Analysis of miRNA Expressions

miRNAs are short endogenous non-coding RNAs of 22 nucleotides in length that negatively regulate gene expression through base pairing with target mRNAs [32]. Deregulation of miRNA and miRNA-related genetic alternations are involved in causing cancers [33]. To unravel the pattern of differential regulation of miRNA, expressions of 534 miRNAs including 470 human miRNAs were profiled in 240 tumor tissue samples and 10 normal tissue samples by Agilent 8 × 15 K Human miRNA-specific microarrays. A total of 149 miRNA were differentially expressed between the GBM tissue and normal tissues samples which were identified by Wilcoxon rank-sum test (P-value for declaring significance after Bonferroni correction is 9.36 × 10^-5^). Of the 149 differentially expressed miRNA, 73 miRNAs were up regulated and 76 down regulated (Additional files 4). Among them, 21, 81 and 15 miRNAs were reported to be associated with the GBM, other cancers and other diseases, respectively, in the literatures [34].

**Figure 4:**
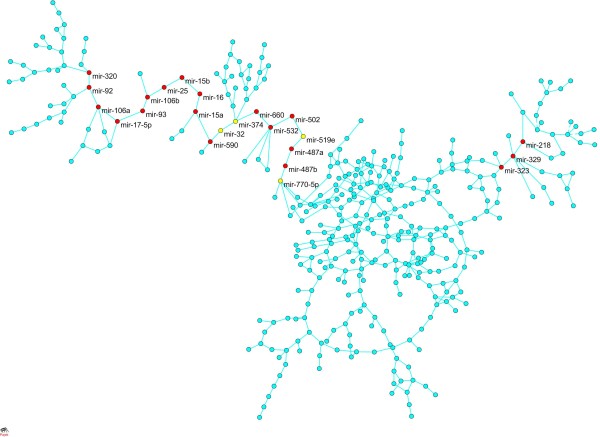
**MiRNA coexpression network**. The largest connected miRNA coexpression network with the average shorted path 10.75 and diameter 49 had 385 miRNAs and 451 edges. MiRNAs with damage values larger than 20 were highlighted in red and yellow, where red nodes denoted significantly differentially expressed miRNAs (P-value < 9.36 × 10^-5^) and yellow nodes denoted they were not significant.

Similar to genes, miRNA are not isolated, instead they act together to perform biological functions [35,36]. To understand how miRNA regulate biological processes at a system level, we used Lasso algorithms for a Gaussian graphical model to map the simultaneous expression of miRNA into a coexpression network in which nodes represent miRNA and edges represent conditional dependence between two connected miRNAs, given all other miRNA expressions in the network (see Methods). The largest connected miRNA coexpression network with the average shortest path (10.75) and diameter (49) had 385 miRNAs and 451 edges (Figure 4). One main feature of this miRNA coexpression network is that mir-770-5p and mir-329 divided the whole network into three subnetworks, the left, middle, and right subnetwork. The middle subnetwork was densely connected.

Similar to the gene coexpression network, we use the damage value of a node as a robustness measure to rank the importance of a miRNA in the miRNA coexpression network. We ranked all differentially expressed miRNA in the largest connected miRNA coexpression network according to their damage values. The top 19 differentially expressed miRNAs with the largest damage values (> 20) which are referred to as fragile miRNAs) were listed in Additional files 5. Of the 19 fragile miRNAs, 16 miRNAs (14 miRNAs were over expressed in the GBM tissues) were in the left coexpression subnetwork and 3 under expressed miRNAs were in the right subnetwork. The middle subnetwork contains no fragile miRNAs. Figure 4 shows that miRNAs in the middle subnetwork were densely connected. The middle network with large numbers of redundant miRNAs was highly robust in response to perturbation of external forces. Mir-487a, mir-487b, mir-502 and mir-532 were the most important components in the miRNA coexpression network. Removal of one of them would cause disconnection between the left part and right part of the miRNA coexpression network and hence lead to dysfunction of the whole miRNA coexpression network. We observed that mir-487a and mi-487b were down regulated, and mir-502 and mir-532 were up regulated in the GBM tissues. SNPs within the miR-502 seed binding region in the 3'-UTR of the SET8 gene which methylates TP53 is reported to be associated with early age of onset of breast cancer [37]. Their major functions are to regulate cell cycle, DNA replication, cytokine-cytokine receptor interaction, hematopoietic cell lineage and signal transduction (see details in the next section). It is interesting to note that three of the 19 fragile miRNAs were significantly related to GBM survival time (univariant cox regression [38]). The three fragil miRNAs include hsa-mir-487b with P-value: 0.0063 and hazard ratio: 1.349; hsa-mir-17-5p with P-value: 0.0035 and hazard ratio: 0.743; and hsa-mir-106a with P-value: 0.0155, hazard ratio: 0.791. Therefore, we predict that these miRNAs are cancer related and play an important role in tumorigenesis and survival.

### miRNA Target Networks

miRNAs down regulate gene expressions by base-pairing with the 3'-noncoding region of the target mRNAs. It is estimated that up to 30% of the genes might be regulated by miRNAs [39]. It is hypothesized that miRNAs and their targets form complex networks to perform various biological functions. To reveal mechanisms of the GBM, we identified target genes of the miRNAs and constructed miRNA target networks. Procedures for discovering the target genes consisted of two steps. The first step was to conduct sequence analysis that used sequence complementarities of miRNA and its target site to predict potential miRNA target genes. Since miRNAs repress the expression of its target gene, the second step is to test the inverse relationship between the expression profile of the miRNA and that of its potential target gene.

To achieve this, we regressed the expression of target mRNA on the expression of miRNAs and selected mRNA with significant negative regression coefficients as miRNA targets. The P-value for declaring significant evidence of the miRNA target was *1.00 *× *10^-4^*. We also searched the predicted potential miRNA targets in the miRGen [40], which integrated animal miRNA targets according to combinations of four widely used target prediction programs (miRanda, PicTar, TargetScan, and DIANA-microT), and experimentally supported targets from TarBase [41] and miR2Disease [34].

The above methods were applied to miRNA and mRNA expression dataset in 237 tumor tissue samples and 10 normal tissue samples with 1,697 differentially expressed mRNAs and 149 differentially expressed miRNAs. This resulted in extremely complex miRNA target networks. We found 3,953 matched miRNA-mRNA pairs for 127 differentially expressed miRNAs and 1,089 differentially expressed genes. Of the 3,953 target pairs, 65 down regulated miRNA targets 468 over expressed genes while 62 up regulated miRNAs target 621 under expressed genes (Additional files 6).

Among them, a total of 14 previously verified targets of 8 miRNAs by experiments elsewhere were listed in Table 4. Of 8 miRNAs, 4 under expressed miRNAs (mir-124a, mir-29b, mir-29c and mir-33) function as a tumor-suppressor and 4 over expressed miRNAs (mir-155, mir-16, mir-21 and mir-210) function as an oncogene (Table 4). CTDSP1 has been reported to be a validated target gene of mir-124a [42], RTN4 and SLC25A22 were validated targets of mir-16 [43]. mir-21 was found to be over expressed in multiple cancers down regulated tumor suppressor genes (TPM1, PTEN, PDCD4 BASP1 and RTN4) in invasion and metastasis of cancer [44]. BASP1 is a transcriptional co-suppressor for the Wilms tumor suppressor protein WT1, thus it can regulate WT1 transcriptional activity [45]. Nogo-A, one protein isoforms encoded by RTN4, turned out to be a neuronal protein involved in diverse processes that goes from axonal fasciculation to apoptosis [46]. Mir-155 targeted a regulator of the apoptosis gene LDOC1 [47]. Up-regulation of miR-210 directly targeted gene EFNA3, which is crucial for endothelial cell response to hypoxia, affecting cell survival, migration, and differentiation [48]. Mir-29c was reported to be under expressed in nasopharyngeal carcinomas and up regulated genes COL4A1, COL4A2 and TDG [49]. COL4A1 and COL4A2 were genes encoding extracellular matrix proteins, as previously discussed; they play an important role in apoptosis, proliferation regulation and anticancer drug resistance [24]. COL4A2 was validated to be also targets of mir-29b in another research group [50]. TDG was involved in DNA repair, a process frequently dysregulated in many cancers [49]. In mouse and human cells, miR-33 inhibits the expression of ABCA1, thereby attenuating cholesterol efflux to apolipoprotein A1 [51]. It was also reported that the role of miR-33 controlling the hematopoietic stem cells self-renewal through p53 may lead to the prevention and treatment of hematopoietic disorders [52].

**Table 4:** Experimentally verified targets.

miRNA	P-value for differential expression of miRNA	Up or down regulated miRNA	Gene	P-value for differential expression of gene	Up or down regulated gene	Regression coefficient	P-value for testing association of miRNA with target mRNA	Coefficient of determination	Function of gene	References
mir-124a	8.62E-08	Down	CTDSP1	8.4E-07	Up	-17.7	2.22E-16	0.244		[42]
mir-155	1.31E-07	Up	LDOC1	1.67E-07	Down	-197	4.00E-15	0.223	tumor suppressor	[47]
mir-155	1.31E-07	Up	SCAMP1	3.27E-06	Down	-30.9	6.06E-10	0.145		[43]
mir-16	0.0000178	Up	RTN4	9.44E-08	Down	-766	2.66E-08	0.119	tumor suppressor	[43]
mir-16	0.0000178	Up	SLC25A22	3.36E-08	Down	-37.8	1.97E-11	0.168		[43]
mir-16	0.0000178	Up	VTI1B	7.15E-08	Down	-122	1.03E-05	0.0765		[43]
mir-21	9.98E-08	Up	BASP1	2.98E-08	Down	-743	0.00E+00	0.349		[44]
mir-21	9.98E-08	Up	RTN4	9.44E-08	Down	-246	1.16E-05	0.0756		[44]
mir-210	1.48E-06	Up	EFNA3	4.67E-07	Down	-10.4	4.61E-05	0.0657		[48]
mir-29b	2.07E-07	Down	COL4A2	1.83E-07	Up	-164	8.92E-05	0.0609		[50]
mir-29c	3.66E-07	Down	COL4A2	1.83E-07	Up	-260	1.99E-07	0.105		[49]
mir-29c	3.66E-07	Down	COL4A1	4.91E-08	Up	-389	1.35E-08	0.124		[49]
mir-29c	3.66E-07	Down	TDG	5.21E-07	Up	-95.5	3.22E-14	0.21		[49]
mir-33	9.6317E-06	Down	ABCA1	1.0396E-06	Up	-114.5	5.941E-12	0.176		[51]

The resulting miRNA target networks have several remarkable features. First, many important genes in the mRNA coexpression networks were targets of differentially expressed miRNAs. Through our prediction, we found that the top 17 genes with damage values greater than 19 in the gene coexpression network were negatively regulated by 34 differentially expressed miRNAs (Additional files 7). All 17 genes and many miRNAs were crucial components in the gene and miRNA coexpression networks. Their altered expressions played an important role in tumorigenesis. Among them, over expressed *RBBP4 *(P-value < 1.8 × 10^-8^) with the largest damage value (37) in the table was negatively regulated by under expressed mir-29b (P-value < 2.07 × 10^-7^) and mir-29c (P-value < 3.66 × 10^-7^). Under expressed *TRIM8 *(P-value <*3.77 *× *10^-6^*) with a damage value of 29 was negatively regulated by over expressed mir-629 (P-value < 2.02 × 10^-7^). Over expressed *TP53 *(P-value <*1.40 *× *10^-7^*) with a damage value of 23 was negatively regulated by underexpressed mir-485-5p. *RBBP4 *has been implicated in chromatin remodeling and regulation of cell proliferation. It has been reported that *RBBP4 *is overexpressed in different human tumors, such as lung, liver and thyroid cancer, acute myelocytic leukemia, and acute lymphoblastic leukemia [53]. *TRIM8 *is thought to be a new tumor suppressor gene [54].

**Figure 5:**
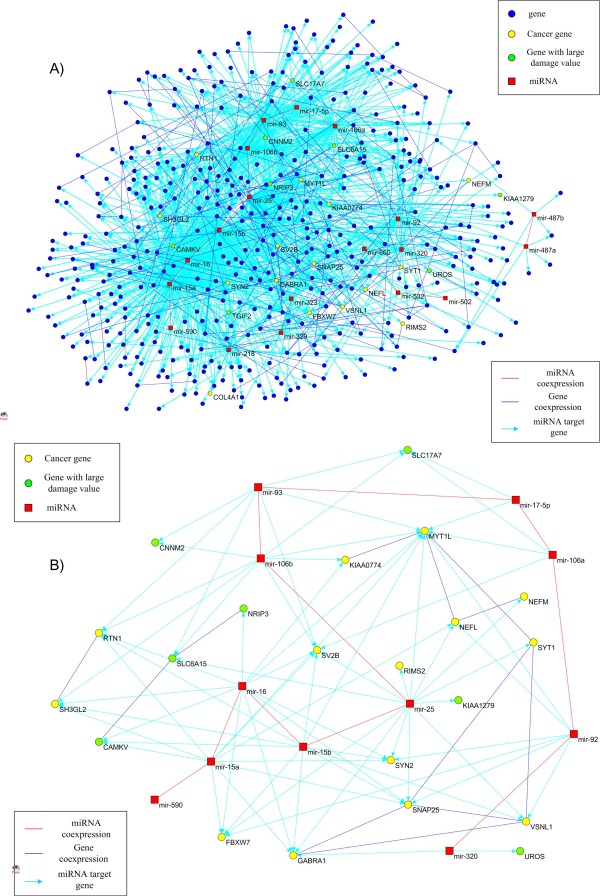
**MiRNA target (sub)network**. (A) To study the function of the top 19 differentially expressed miRNAs with the largest damage values, we constructed mRNA target networks of these 19 miRNAs with 476 nodes and 1,128 arcs and 174 edges, where 5 underexpressed miRNAs negatively regulated 85 overexpressed genes and 14 overexpressed miRNA negatively regulated 372 underexpressed genes. 1128 arcs were miRNA-mRNA pairs, 15 edges connected coexpressed miRNAs, and remaining 159 edges linked coexpressed genes. The genes with large damage value or related to cancer in the co expression network genes were highlighted and those gene names were marked in the figure. (B) MiRNA target subnetwork. 7 genes with the damage values greater than 15 and 14 cancer related genes were regulated by 11 miRNAs with the damage values greater than 20 (Additional files 12). Those nodes and edges were extracted from Figure 5A mRNA target network.

Second, many genes targeted by critical components in the miRNA coexpression network, played important roles in the regulation of neural process and tumor genesis. To study the function of the top 19 differentially expressed miRNAs with the largest damage values (Additional files 5), we constructed mRNA target networks of these 19 miRNAs with 476 nodes and 1,128 arcs and 174 edges as shown in Figure 5(A), where 5 under expressed miRNAs negatively regulated 85 overexpressed genes and 14 overexpressed miRNA negatively regulated 372 underexpressed genes, 1,128 arcs were miRNA-mRNA pairs, 15 edges connected coexpressed miRNAs, and the remaining 159 edges linked coexpressed genes (Additional files 8). The genes with large damage value or related to cancer in the gene coexpression network (Additional files 3) were highlighted and marked in Figure 5(A). We extracted two subnetworks from Figure 5(A). The first subnetwork consisted of 8 genes (*KIAA1279, CNNM2, CAMKV, TGIF2, SLC6A15, SLC17A7, NRIP3*, and *UROS*) with damage values greater than 15 which were regulated by 11 miRNAs (mir-25, mir-106b, mir-93, mir-15a, mir-16, mir-15b, mir-329, mir-218, mir-17-5p, mir-106a, and mir-320) with damage values greater than 20. The second subnetwork consisted of 14 under expressed cancer genes (*FBXW7, GABRA1, MYT1L, NEFL, NEFM, SNAP25, SYT1, VSNL1, RTN1, SH3GL2, SV2B, SYN2, KIAA0774 *and *RIMS2*) which were regulated by 10 over expressed miRNAs (mir-15b, mir-25, mir-16, mir-92, mir-15a, mir-320, mir-106b, mir-93, mir-106a, and mir-17-5p) in the mRNA coexpression network. Two subnetworks are shown in Figure 5(B). Two subnetworks are involved in the synaptic transmission process. Neuron communication occurs at the synapse via neurotransmitters. *FBXW7 *serves as a negative regulator of oncoprotein and is a general tumor suppressor. It has been reported that *FBXW7 *is implied in various cancers including glioblastoma [55]. *GABRA1 *encodes a gamma-aminobutyric acid (GABA) receptor and *GABA *is the major inhibitory neurotransmitter in the mammalian brain. *MYT1L *(myelin transcription factor 1-like) regulates nervous system development. Both *NEFL*and *NEFM *are neurofilaments. They play a role in intracellular transport to axons and dendrites. SNAP25 is a presynaptic plasma membrane protein and regulates neurotransmitter release. SNAP25 was reported to be implicated in neuritogenesis in human neuroblastoma [56]. Both SYT1 and VSNL1 serve as Ca(2+) sensors in synaptic transmission [16]. SYN2 is a member of the synapsin gene family, SV2B is a synaptic vesicle glycoprotein and RIMS2 regulates synaptic membrane exocytosis [16].

Table 5 summarizes the major functions of target genes of the miRNAs in Figure 5(A) where one-sided Fisher's exact test was used to test for significantly enriched GO categories or pathways. Table 5 shows that the target genes of these miRNAs were mainly involved in cancer related signaling pathways and nervous system processes including synapse, synaptic transmission, neurotransmitter transport, nervous system development and neurological system processes. It is interesting to note that coexpressed mir-15a, mir-15b, mir-16, mir-25 and mir-92 were major miRNAs that targeted genes in both cancer related signaling pathways and nervous system processes, and mir-16a was significantly associated with the GBM survival time.

**Table 5:** Function of target genes of 9 miRNAs.

Gene Ontology (GO) category or pathway	Enrichment P-values								
	
	mir-15b	mir-15a	mir-16	mir-25	mir-106a	mir-93	mir-106b	mir-92	mir-323
Glioma		1.9E-02							
Regulation of neurotransmitter levels		1.3E-02	1.1E-02						
Neurotransmitter transport	6.8E-03	1.5E-02	3.3E-02	3.9E-02					
Long-term potentiation	4.2E-03	3.7E-05	2.4E-03	3.1E-02		1.4E-02	5.3E-03		
Synaptic transmission	2.4E-04	4.0E-04	5.3E-04	3.0E-06		4.4E-02		1.6E-03	
Neurotransmitter secretion		1.7E-02	1.0E-02	2.9E-02					
Nervous system development	3.2E-02	1.3E-02	5.5E-03				2.7E-02		
Central nervous system development			2.5E-02						
Transmission of nerve impulse	2.3E-04	9.6E-04	4.8E-04	1.8E-06				6.0E-04	
Regulation of synaptic plasticity				4.6E-02					
Synaptic vesicle transport		2.8E-02						3.1E-02	
Regulation of synapse structure and activity				4.6E-02					
Neurological system process	7.1E-03	4.3E-03	5.1E-03	1.5E-04				3.1E-03	
Learning and/or memory	3.7E-02								
Learning	4.0E-02								
Neurofilament				2.1E-02					
Synaptic vesicle	1.1E-02		8.0E-03	2.7E-02					
Synaptosome								1.4E-02	
Synaptic vesicle membrane				3.4E-02					
Neuron projection			4.5E-02						
Synapse part				4.7E-05	1.5E-02	2.5E-03	1.6E-02	1.2E-02	
Synapse	4.9E-03	1.5E-02	2.1E-02	1.1E-07	6.2E-03	8.4E-04	5.3E-03	2.3E-04	
Axon guidance						2.1E-02			
Antigen processing and presentation									4.0E-02
Ras protein signal transduction					1.9E-02		2.3E-02		
Calcium ion transport	1.8E-03	3.6E-04	7.8E-03				4.2E-02		
Calcium signaling pathway	2.7E-04	7.5E-06	1.7E-03	1.7E-02		2.0E-04	2.2E-05		
Cell communication	1.9E-02	2.2E-02	5.6E-03	2.3E-03		1.8E-02	2.6E-02	2.5E-02	
Cell-cell signaling	1.4E-03	8.1E-04	9.5E-04	4.2E-06				7.0E-03	
Wnt signaling pathway		4.0E-02				2.9E-03	7.6E-03		
MAPK signaling pathway		1.5E-02	8.4E-03			3.8E-02	2.6E-02		
GnRH signaling pathway	1.0E-02	8.7E-05	6.6E-03						
ErbB signaling pathway		2.2E-02	3.0E-02						
B cell receptor signaling pathway						6.3E-03			
VEGF signaling pathway						8.4E-03			
T cell receptor signaling pathway						2.1E-02			
Apoptosis						2.1E-02			

Third, the contribution of miRNAs to the expression variation of some genes is very high. The proportion of expression variation of target genes explained by the linear influence of miRNA variation can be measured by the coefficient of determination (*R*^2^). In Additional files 9, there were a total of 78 under expressed genes with the coefficient of determination greater than 20% which was negatively regulated by 7 over expressed miRNAs (mir-15a, mir-15b, mir-16, mir-25, mir-93, mir-106a and mir-106b). Surprisingly, up to 33% of the expression variations of the under expressed gene *GCC2 *were explained by a single over expressed mir-25. Functional roles of *GCC2 *in cancer are unknown. The gene with the second largest coefficient of determination was *CAMK2N1*. Over expressed mir-106b explained 28% of the expression variations. *CAMK2N1 *is reported to be a candidate tumor suppressor [57]. The function of *CAMK2N1 *is to inhibit MEK/ERK activity and induce p27 accumulation which negatively regulates cell-cycle progression. Reducing expression of *CAMK2N1 *will accelerate tumor growth. 26% of the expression variation of *CPEB1 *that regulates synaptic plasticity and is implied in cancer development [58] and *GRM1 *that is involved in neurotransmitter in the central nervous system and implicated in melanoma [59] was explained by overexpressed mir-25 and mir-15b, respectively. Expressions of genes *SYT1, SNAP25, GABRA1, VSNL1, MYT1L, SV2B, SYN2 *and *SLC12A5 *which were involved in synaptic transmission, were also largely regulated by miRNAs.

### Decipher the Path Connecting Mutations, Expression and the GBM

Unveiling the path from mutations to tumor formation will help us to uncover the mechanisms of the cancer. Mutations often cause the formation of the tumor through their contributions to the variation of gene expressions or miRNAs in the regulatory or signal transduction networks which in turn influence the development of the tumor [4,60]. Interestingly, some genes harboring somatic mutations that were associated with GBM were also differentially expressed. Genes *TP53 *(P-value <1.40 × 10^-7^), *RB1 *(P-value < 1.24 × 10^-7^), and *COL3A1*(P-value < 1.55 × 10^-6^) were significantly differentially expressed. *MKI67 *(P-value < 7.73 × 10^-5^), *CHEK2 *(P-value < 4.30 × 10^-5^), *GSTM5 *(P-value < 1.30 × 10^-5^), *BCL11A *(P-value < 1.12 × 10^-5^) and *FN1*(P-value < 8.19 × 10^-6^) were mildly differentially expressed. *TP53 *with a damage value of 23 and *COL3A1 *with a damage value of 21 played a crucial role in the gene coexpression networks.

To study the function of somatic mutations and LOH and connect them with disease through gene expressions and miRNAs, we studied their *cis *or *trans *regulatory effects on gene or miRNA expression traits. Since the popular methods for eQTL analysis have mainly focused on testing *cis *or *trans *regulatory effects individually, their applications to testing *cis *or *trans *regulatory effects of rare somatic mutations and LOH are inappropriate. We applied the group regression method to expression profiles of 12,043 genes produced by Affymetrix HT Human Genome U133 Array Plate Set at MIT [3] and expression profiles of 470 human microRNAs produced by Agilent 8 × 15KHuman miRNA-specificmicroarray at the University of North Carolina in 169 tumor tissue samples from glioblastoma patients [3], which were shared among the gene expression, miRNA expression and mutation datasets.

We first studied the *cis *regulatory effects of somatic mutations and LOH on mRNA or miRNA expression traits. For somatic mutation, we found four cis-eQTL (*TP53, EGFR, NF1 *and *PIK3C2G*). P-values for testing association of somatic mutations in *TP53, EGFR, NF1 *and *PIK3C2G *with their expressions were 0.033, 0.019, 0.028 and 0.006, respectively. Fold change (defined as the ratio of their average expressions of the samples with somatic mutations over the average expressions of the samples without somatic mutations) for the 4 genes were 1.10, 1.73, 0.86 and 1.23, respectively. Although regression analysis did not show significant association of somatic mutations in *BCL11A, FN1 *and *COL3A1 *with their expressions due to very low frequencies of mutations, the fold change were 1.45, 0.60 and 0.20, respectively. We still observed some regulatory effects of somatic mutations on these two genes. For LOH mutation, two cis-eQTL (*NRAP *and *EGFR*) were detected with P-values 0.030 and 0.034 and fold changes 0.90 and 2.32, respectively. Regression analysis did not show significant association of LOH mutations in the gene CYP1B1 with their expressions, but the fold change was 0.48.

**Figure 6:**
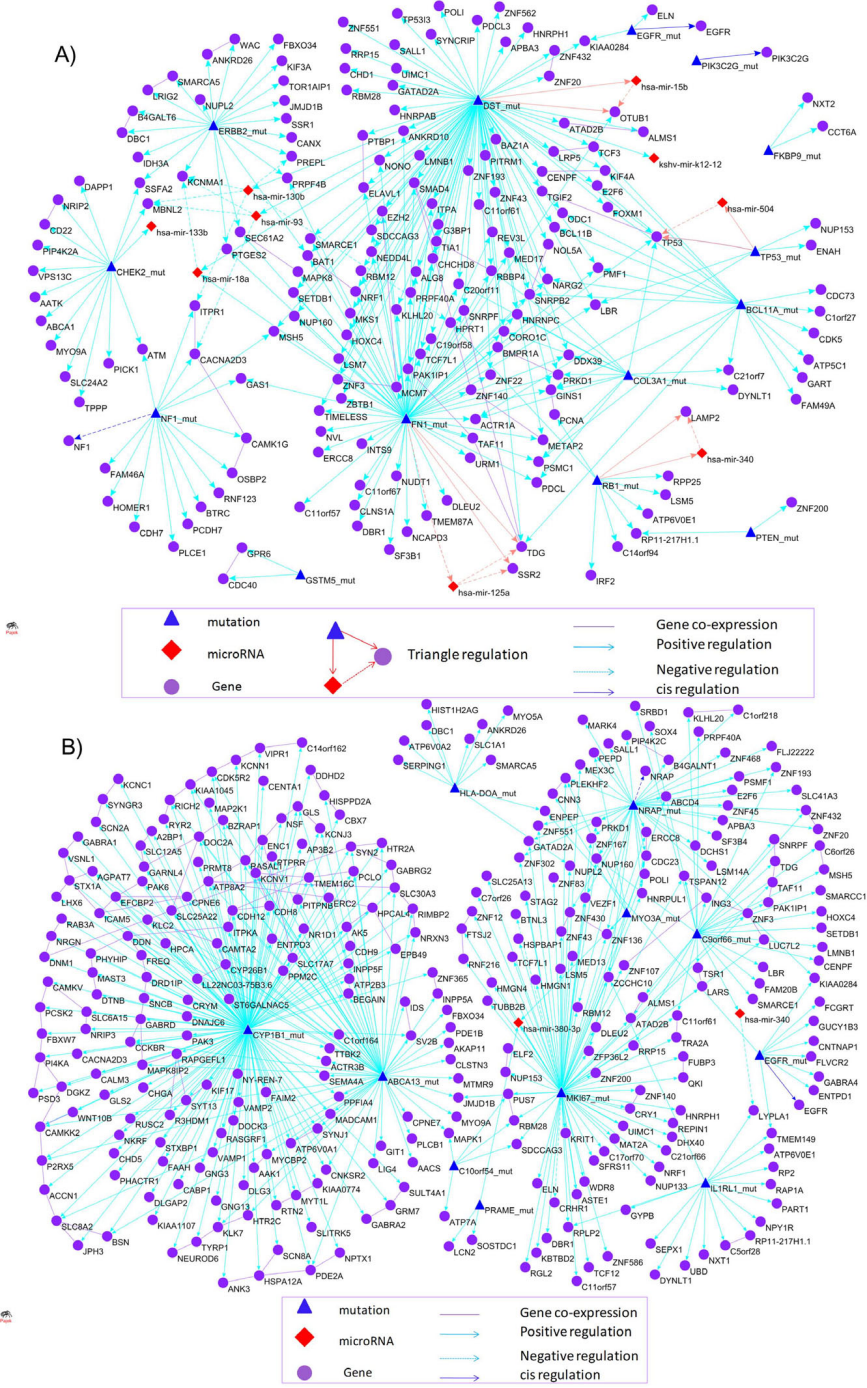
**Somatic and LOH mutation eQTL network**. (A) Somatic mutation eQTL network. A network that connects 14 genes with somatic mutations associated with GBM, their regulated mRNAs and miRNA expressions was shown. (B) LOH mutation eQTL network. A network that connects 11 genes with LOH associated with GMB, their regulated mRNAs and miRNA expressions.

Next we identify the *trans*-regulatory effects of somatic mutations and LOH on mRNA or miRNA expression traits. The thresholds for declaring significant association of the set of somatic mutation and LOH in the gene with mRNA and miRNA expression after Bonferroni correction for multiple tests were 1.63 × 10^-4 ^and 4.03 × 10^-4^, respectively. A network that connects 14 genes with somatic mutations associated with GBM, and their regulated mRNAs and miRNA expressions is shown in Figure 6(A). Somatic mutations in these 14 genes were strongly correlated with expressions of 177 significantly differentially expressed genes, 45 of which were under expressed and 132 were over expressed in tumor tissue samples, a total of 262 trans gene eQTL were found (Additional files 10). These mutations also significantly affected expressions of 23 miRNAs, 11 of which were underexpressed and 12 were overexpressed and a total of 26 trans miRNA eQTL were found (Additional files 11). Remarkably, we found 5 paths (Table 6): (1*) RB1 *with association of somatic mutations with GBM regulated expressions of both gene *LAMP2 *and mir-340, and mir-340 in turn targeted gene *LAMP2*; (2) DST with association of somatic mutations regulated expressions of both gene *OTUB1 *and mir-15b, and mir-15b in turn targeted gene *OTUB1*; (3) FN1 with association of somatic mutations regulated expressions of both SSR2 and mir-125a, and mir-125a in turn targeted gene *SSR2*; (4) *FN1 *with association of somatic mutations regulated expressions of both gene *TDG *and mir-125a, and mir-125a in turn targeted gene *TDG*; and (5) *TP53 *with association of somatic mutations regulated expressions of both gene *TP53 *and mir-504, and mir-504 in turn targeted TP53. Similarly, Figure 6(B) shows a network that connected 11 genes with LOH associated with GMB, their regulated mRNAs and miRNA expressions. These 11 genes with LOH as *trans*-eQTLs affected 323 differentially expressed or interacted genes, 190 of which were under expressed and 133 were over expressed, a total of 409 *trans gene*-eQTL were found (Additional files 12). The LOH also affected expressions of 19 miRNAs, 9 of which were underexpressed and 10 were overexpressed, and a total of 27 *trans miRNA*-eQTL were found (Additional files 13).

**Table 6:** Genes with somatic mutations regulate both mRNA and miRNA expressions, and formed triangle regulation cycles.

Mutation	Gene expression				miRNA expression			
**Gene**	**Gene**	**Damage value**	**P-value for gene expression**	**Up or down regulated**	**miRNA**	**Damage value**	**P-value for miRNA expression**	**Up or down regulated**

**RB1**	LAMP2	0	8.22E-07	Up	hsa-mir-340	0	1.16E-06	Down

**DST**	OTUB1	0	5.33E-07	Down	hsa-mir-15b	45	1.30E-06	Up

**FN1**	SSR2	0	3.67E-07	Up	hsa-mir-125a	2	4.83E-05	Down

**FN1**	TDG	3	5.21E-07	Up	hsa-mir-125a	2	4.83E-05	Down

**TP53**	TP53	23	1.40E-07	Up	hsa-mir-504	1	1.42E-06	Down

**Mutation-gene regression**			**miRNA-gene regression**			**Mutation-miRNA regression**		

**Regression coefficient**	**P-value**	**Coefficient of determination**	**Regression coefficient**	**P-value**	**Coefficient of determination**	**Regression coefficient**	**P-value**	**Coefficient of determination**

1.03E+00	7.04E-04	6.47E-02	-2.77E+02	3.17E-06	8.50E-02	1.81E+00	4.10E-09	1.85E-01

1.23E+00	5.91E-04	6.65E-02	-2.28E+01	9.77E-07	9.34E-02	1.27E+00	3.80E-04	7.18E-02

1.54E+00	1.02E-05	1.07E-01	-2.33E+02	4.92E-05	6.52E-02	-1.18E+00	8.72E-04	6.33E-02

1.39E+00	7.60E-05	8.72E-02	-6.91E+01	4.77E-05	6.54E-02	-1.18E+00	8.72E-04	6.33E-02

2.81E-01	3.30E-02	2.62E-02	-4.14E+01	2.72E-06	8.61E-02	3.43E-01	9.10E-03	3.93E-02

Similar to the differentially expressed gene set enrichment analysis, we searched the enriched GO and pathway groups for the eQTL gene list of every somatic/LOH mutations by DAVID Bioinformatics Resources [31]. The most enriched GO terms included the molecular function term GO:0015075~ion transmembrane transporter activity (for LOH mutated gene *CYP1B1 *eQTL), biological process term GO:0050877~neurological system process (for LOH mutated gene *ABCA13 *eQTL) and cellular component term GO:0005643~nuclear pore (for somatic mutated gene *TP53 *eQTL) with P-values 6.73 × 10^-7^, 4.31 × 10^-5^, and 0.02, respectively. The enriched pathways included the Wnt signaling pathway (for somatic mutated gene *DST *eQTL), colorectal cancer (for somatic mutated gene *FN1 *eQTL) and Gap junction (for LOH mutated gene *ABCA13 *eQTL) with P-values 0.018, 0.012 and 0.013, respectively. Several examples are provided here, for the detailed results, see Additional files 14.

## Discussion

Genetic and molecular alternations that are likely to cause tumor formation are often organized into complex biological networks. The purpose of this report is (1) to explore the possibility of integrating altered DNA variations, mRNA and miRNA expression variations into multi-level complex genetic networks that contribute to tumorigenesis; (2) to decipher paths from somatic mutations and LOH to tumor formation through genetic and molecular networks; and (3) to identify key genetic alternations causing tumor formation using network approaches. In the first step, we reconstruct single type molecular networks whose components are of the same type of molecules, either mRNAs or miRNAs. We reconstructed mRNA coexpression networks and miRNA coexpression networks for glioblastoma. The coexpression networks attempt to uncover the regulatory relationships among mRNAs or miRNAs. The second step is to reconstruct miRNA target networks that connect mRNA and miRNA coexpression networks and indentify the eQTL network that connects mutations and mRNA, miRNA expression profiles.

We have addressed several issues for deciphering the paths from somatic mutations and LOH to tumor formation. The first issue is how to test association of somatic mutations or LOH with glioblastoma. We developed a group association test that is based on population genetics for assessing association of somatic mutations and LOH with cancer. We identified 14 genes harboring somatic mutations associated with GBM and 11 genes harboring LOH associated with glioblastoma. The second issue is how to uncover the components of mRNA or miRNA coexpression networks which respond to somatic mutations and LOH associated with glioblastoma. Traditionally, when alleles are common, these components are identified by mapping *cis*-eQTL or *trans*-eQTL. However, when alleles are rare, these components are hard to find by individually mapping *cis*-eQTL or *trans*-eQTL. The approach developed here is to extend group tests for a qualitative trait to a quantitative trait. The components that respond to perturbation of rare somatic genetic variants were identified by regressing the expression of an mRNA or a miRNA on the number of all mutated alleles across the gene. We discovered large comprehensive genetic and molecular networks that connect genes harboring associated mutations or LOH, mRNAs and miRNAs. Interestingly, we found five triangle cycles in the networks which indicated that significantly associated somatic mutations or LOH regulated both differentially expressed mRNA and miRNA and the miRNA in turn also affected expression levels of the mRNA. The approach presented here has two remarkable features. First, it offered a powerful tool for differentiating driver mutations from passenger mutations. Second, it provides functional information on how somatic mutations or LOH lead to tumorigenesis through complex genetic and molecular networks.

Our studies support the hypothesis that cancer may be the emergent properties of many genetic variants that are highly interconnected. Genetic and environmental stimuli can be viewed as random attacks to genetic and molecular networks. Topological properties of the genetic and molecular networks are closely related to the function of cells. Cancer arises from the failure of networks to respond to attacks. In other words, the attacked networks are unable to return to their normal states and remain functional in the face of random perturbations. We suspect that key components of a network contributing to the robustness of the network also play an important role in the function of the cells. We modeled over or under expression of mRNAs and miRNAs, and genetic alternations as deletions of a node in the network which will cause dynamical changes in the network and used a damage value of the node to measure its contribution to the robustness of the network. We found that several cancers related genes such as *TP53 *have large damage values in the genetic and molecular networks. These key components in the network may serve as therapeutic intervention points.

Our results are preliminary. Although network analysis may have the potential to unravel the mechanism of tumor initiation and progression, the presented network structures and their properties in this report may depend on sampled tissues. Whether the structures of our reconstructed network can be replicated in other tissues or not is the key to the success of network analysis in cancer studies.

## Conclusion

In this paper, we use system biology and network approaches to develop novel analytical strategies for (1) systematically integrating altered DNA variations, mRNA and miRNA expression variations into multi-level complex genetic networks that contribute to tumorigenesis, (2) deciphering paths from somatic and LOH mutations to tumor formation through genetic networks and (3) identifying key genetic alternations causing tumor formation by network analysis.

## Methods

### Test Association of Somatic Mutations and LOH with Glioblastoma

Cancers arise from mutations that confer growth advantage on cells [61]. The somatic mutations in cancers can be classified either as "drivers" or "passengers" [62]. In other words, mutations often have no effect on the development of a tumor. As the number of tumor tissues and normal tissues increases we can observe somatic mutations in both tumor and normal tissues. The current popular method for identifying driver mutations is to compare the difference in the mutation rates [4,63]. However, there is debate about how to assess a significant excess of mutations in tumors [64]. We need to develop formal tests to detect differences in mutation rates between tumor and normal tissues. Most traditional statistical methods that often test the association of genetic variants individually were designed for testing association of common alleles with common diseases and are inappropriate for testing the association of rare somatic mutations. A feasible approach is to record rare sequence variants at different genome positions and to collectively test the association of a set of rare variants. It has been shown that the number of rare alleles in large samples is approximately distributed as a Poisson process with its intensity depending on the total mutation rate [15]. The intensity of the Poisson process within a segment of the genome can be interpreted as the mutation rate. Similar to the standard χ^2 ^test for association of SNPs which compare the differences in allele frequencies between cases and controls, the proposed statistics are to compare difference in the mutation rates between tumor and normal samples. Specifically, let $\bar{U}$ and $\bar{V}$ be the average number of rare mutations in a gene which is the intensity of the Poisson process underlying the rare variants, in the tumor and normal samples in one specific gene, respectively. Let *S_uv _*be the pooled sample variance of the rare variants. Define the test statistic: $T_{G}=\frac{{\left(\bar{U}-\bar{V}\right)}^{2}}{(\frac{1}{n_{A}}+\frac{1}{n_{G}})S_{uv}}$, where *n_A _*and *n_G _*are the number of sampled tumor tissues and normal tissues, respectively. Under the null hypothesis of no association of the set of rare variants with the disease, the average number of rare alleles in cases and controls should be equal and the statistic *T_G _*is asymptotically distributed as a central $\chi_{\left(1\right)}^{2}$ distribution. In some cases, we may have a homozygous genotype of rare mutations. To improve the power, in this case, we can count it twice. Instead of defining statistics in terms of genotype, we can similarly define the test statistics in terms of the rare alleles, which is denoted as *T_a_*. The statistic *T_a _*counts the number of mutated alleles at the locus. Thus, it will count homozygote of rare variants twice.

To examine the validity of the test statistics, we performed a series of simulation studies. We used infinitely many allele models and software (GENOME) to generate rare variants. Suppose that the mutation rate per generation per base pair is 1.00 × 10^-8^, the recombination rate between consecutive fragments is 0.0001, and the migration rate per generation per individual is 0.00025. We simulated 100 fragments of which each fragment length equals 10k base pair. A total of 100,000 individuals who were equally divided into cases and controls were generated in the general population, 500-2,000 individuals were randomly sampled from each of the cases and controls and 10,000 simulations were repeated. Table 7 summarizes the type I error rates of two statistics. Table 7 shows that the estimated type I error rates of the statistics for testing association of a set of rare variants with the disease were not appreciably different from the nominal levels α = 0.05, α = 0.01 and α = 0.001.

**Table 7:** Type 1 error rates of the statistics *T_G_*, *T_a_*.

Sample sizes		*T_G_*	*T_a_*
2,000	α = 0.001	0.0012	0.001
	α = 0.01	0.0093	0.0096
	α = 0.05	0.0489	0.0494

1,500	α = 0.001	0.0015	0.001
	α = 0.01	0.0106	0.0092
	α = 0.05	0.0521	0.0500

1,000	α = 0.001	0.0012	0.0007
	α = 0.01	0.0106	0.0097
	α = 0.05	0.0522	0.0459

500	α = 0.001	0.0009	0.0008
	α = 0.01	0.0102	0.0117
	α = 0.05	0.0504	0.0548

A LOH mutation was recorded when the genotype in blood or normal tissue is heterozygous, and in the tumor tissue, the reference allele loses normal function and the genotype becomes homozygous. The statistic *T_G _*can also be used to test association of LOH with glioblastoma.

### Survival Analysis Links Gene and microRNA Signatures with GBM Survival Time

Patients (n = 358) with complete clinical information were obtained from the TCGA data portal. Survival analysis is used to deal with these time-to-event censored data. Censoring refers to the patients who may drop out or are still alive at the end of the study. In fact, leaving censored patients out would introduce bias to the remaining uncensored samples, and it is difficult to make adjustments for such bias. The approach we use, Cox proportional hazard regression, is a standard method in biostatistics for dealing with survival data [38]. For microRNA signature, we use the univariate Cox proportional hazard regression model to regress the survival time on every microRNA [65]. Hazard ratios from the Cox regression analysis were used to identify which microRNA signatures were associated with death from the recurrence of cancer or any cause. Protective signatures were defined as those with a hazard ratio for death < 1. High-risk signatures were defined as those with a hazard ratio for death > 1. All analyses were done with the SAS version 9.1 software (SAS Institute Inc). Two-tailed tests and P-values < 0.05 for significance were used.

### Lasso for Coexpression Networks

A co-mRNA expression or co-miRNA expression network can be constructed by joint sparse regression for estimating the concentration matrix in which off-diagonal elements represents the covariance between the corresponding variables conditional on all other variables in the network [66]. Sparse regression for reconstruction of coexpression network is briefly introduced here. (For details, see[66]). Denote the mRNA or miRNA expression levels as variables *y*_1_,...*y_q_*. A variable is represented by a node. An edge connecting two nodes indicates that the connected two variables are conditionally dependent, given all other variables. Assume that the vector of q variables *Y *= [*y*_1_,...*y_q_*]*^T ^*follow a normal distribution *N*(0,Σ). Denote the partial correlations as *ρ^ij ^*= *Corr*(*y_i_*, *y_j_*|*y*_-(*i,j*)_) for 1 ≤ *i *<*j *≤ *q *and -(*i*, *j*) ≡ {*k*:1 ≤ *k *≠ *i*, *j *≤ *q*}. If we assume the normality of the variables, then two variables *y_i _*and *y_j _*are conditionally dependent, given all other variables if and only *ρ^ij ^*≠ 0. Let Σ^-1 ^= (*σ^ij^*) be the concentration matrix. Then, $\rho^{ij}=-\frac{\sigma^{ij}}{\sqrt{\sigma^{ii}\sigma^{jj}}}$. When sample size is much larger than the number of variables, the concentration matrix can be directly estimated from the inverse of the sampling covariance matrix. However, when the number of variables in the network is larger than the sample size, the inverse of the sampling covariance matrix may not exist. We need to develop methods for network modeling via estimating a sparse concentration matrix. One such techniques is sparse regression.

It is well known that the relationship between the partial correlation and regression exists. In other words, if *y_i _*is expressed as $y_{i}=\sum_{j\neq i}\beta_{ij}y_{j}+\varepsilon_{i}$ and *ε_i _*is independent of *y_-i _*then $\beta_{ij}=\rho^{ij}\sqrt{\frac{\sigma^{jj}}{\sigma^{ii}}}$. Therefore the search for nonzero partial correlations can be formulated as a variable selection problem in regression in which the *l*_1 _penalty on a loss function is imposed. Specifically, the sparse regression for estimating a concentration matrix is formulated as

$$L(\beta ,\lambda )=\frac{1}{2}\left(\sum_{i=1}^{q}||Y_{i}-\sum_{j\neq i}\beta_{ij}Y_{j}|{|}^{2}\right)+\lambda \sum_{1\leq i<j\leq q}\left|\beta_{ij}|\right),$$

where *Y_i _*= [*y*_*i*1_,...,*y_in_*]*^T ^*is the sample of the *ith *variable and *λ *is a penalty parameter which controls the size of the penalty. A larger value of *λ *leads to a sparse regression that fits the data less well and a smaller *λ *leads to regression that fits the data well but is less sparse. Let *θ *= [*β*_12,...,_*β*_(*q*-1)*q*_]*^T ^*= [*θ*_1_,...,*θ*_*q*(*q*-1)/2_]^*T*^, $Y={\left[Y_{1}^{T},...Y_{q}^{T}\right]}^{T}$, $X_{\left(i,j\right)}={\left[0,...,0,Y_{j}^{T},0,...,0,Y_{i}^{T},0,...,0\right]}^{T}$ and *X *= [*X*_(1,2)_,..., *X*_(*q*-1,*q*)_]. Then, *L*(*β*, *λ*) can be rewritten as

$$L(\theta ,\lambda )=\frac{1}{2}|{\left(Y-X\theta \right)}^{T}(Y-X\theta )+\lambda \sum_{j}\left|\theta_{j}\right|.$$

Coordinate descent algorithms can be used to minimize *L*(*θ*, *λ*) with respect to *θ*. Let *p *= *q*(*q*-1)/2 Briefly, coordinate descent algorithms are given as follows:

1. Initial step: let (*x*)_+ _= *xI*_(*x *> 0)_, for j = 1,..., p

$$\theta_{j}^{\left(0\right)}=sign(Y^{T}X_{j})\frac{{\left(|Y^{T}X_{j}|-\lambda \right)}_{T}}{X_{j}^{T}X_{j}}.$$

2. Step 2: for j = 1,..., p, update *θ*^(*new*)^⇐ *θ*^(*old*)^:

$$\begin{array}{l} \theta_{i}^{\left(new\right)}=\theta_{i}^{\left(old\right)},i\neq j;\\ \theta_{j}^{\left(new\right)}=sign\left(\frac{e^{\left(old\right)}X_{j}}{X_{j}^{T}X_{j}}+\theta_{j}^{\left(old\right)}\right){\left(\left|\frac{e^{\left(old\right)}X_{j}}{X_{j}^{T}X_{j}}+\theta_{j}^{\left(old\right)}\right|-\frac{\lambda }{X_{j}^{T}X_{j}}\right)}_{+} \end{array}$$

where *e*^(*new*) ^= *Y *- *Xθ*^(*old*)^.

3. We repeat step 2 until converge.

### Functional Module Identification and Gene Set Enrichment Analysis

Coexpression networks are usually organized into functional modules that perform specific biological tasks. Genes within coexpression modules often share conserved biological functions. A dynamic tree cut procedure was used to identify modules [67]. A coexpression network was clustered using hierarchical clustering. Modules are defined as braches of the dendrogram. A one-sided Fisher exact test that calculates the probability of seeing the observed number of genes within a pathway or a GO category in the module by chance was used to test for overrepresentation of a pathway or a GO category in the module. We assembled 465 pathways from KEGG [68] and Biocarta http://www.biocarta.com. To test both the enriched Gene Ontology and pathways for the gene set, for example, the differentially expressed gene list and the eQTL genes of mutations, we used an online tool DAVID (The Database for Annotation, Visualization and Integrated Discovery), which provides a comprehensive set of functional annotation tools for investigators to understand the biological meaning behind large lists of genes [31], http://david.abcc.ncifcrf.gov/.

### Ranking of the Nodes in the Network

Biological functions and mechanisms are encoded in network properties. An important strategy for unraveling the mechanisms of initiation and progression of cancer is to conduct analysis of complex biological networks and study their behaviors under genetic and epigenetic perturbations. Robustness of a biological network, ability to retain much of its functionality in the face of perturbation [9], has emerged as a fundamental concept in the study of network topological properties [10]. Widely used measures of network robustness include ranking the importance of the nodes in the network. One of the most efficient measures of importance in robustness analysis of the network is the damage value of a node which quantifies the effect of the removal of that particular node from the network. Formally, we define the damage value of a node as follows. Let *G *= (*V*, *E*) be the connected component that contains nodes *h *϶ *V*, and let $\tilde{G}=(\tilde{V},\tilde{E})$ be the largest connected component of $\tilde{G}$, after the removal of node *h*. Then, the value $D(h)=|V|-|\tilde{V}|$ is the damage value of node *h*.

### miRNA Target Networks

miRNAs down regulate gene expressions by base-pairing with the 3'-noncoding region of the target mRNAs. Procedures for discovering the target genes consist of two steps. The first step was to conduct sequence analysis that used sequence complementarities of the miRNA and its target sites to predict potential miRNA target genes. We searched the predicted potential miRNA targets in miRGen [40], which integrated animal miRNA targets according to combinations of four widely used target prediction programs (miRanda, PicTar, TargetScan, and DIANA-microT) and experimentally supported targets from TarBase [41] and miR2Disease [34].

Since miRNAs repress the expression of its target gene, the second step is to test the inverse relationship between the expression profile of the miRNA and that of its potential targets. To achieve this, we regressed the expression of the target mRNA on the expression of miRNAs and select the mRNA with significant negative regression coefficients as miRNA targets.

### Identify the Genetic Variants that Have *cis *or *trans *Regulatory Effects on miRNA or mRNA Expression

Loci that are significantly associated with expression are called expression quantitative trait loci (eQTL). Somatic mutations may directly or indirectly regulate the expression of mRNAs or miRNAs. The traditional statistical methods to regress the expression levels on the individual genetic variant for identifying the eQTL are inappropriate for studying the regulatory effect of somatic mutations due to their low allele frequencies. An alternative approach to the current variant-by-variant regression method is groupwise regression methods in which a group of rare genetic variants are jointly analyzed. It is well known that eQTL includes *cis*-eQTL in which an association exists between the expression of a specific gene (mRNA) and the genetic variants at that gene's locus, or between the expression of miRNA and the genetic variants at its precursor miRNA, and *trans*-eQTL in which there is an association between the expression of a gene or a miRNA and the genetic variants at a non-local genomic locus. Regression methods that regress the expression of an mRNA or an miRNA on the number of all mutated alleles across the region of interest were used to identify *cis*- or *trans*-eQTL.

## Authors' contributions

H Dong analyzed most of the datasets, constructed the miRNA target network and eQTL network and wrote the paper; L Luo wrote the program for the mutation group association test and identifying eQTLs by regressing mRNA or miRNA on all somatic mutations or LOH within the gene; S Hong was responsible for the gene and miRNA coexpression network analysis; H Siu analyzed the eQTL network; Y Xiao wrote programs for the node importance ranking algorithm; L Jin and R Chen provided advice on the project and revised the drafts; M Xiong designed the project and wrote the paper. All authors contributed to the writing and approved the final manuscript.

## Supplementary Material

Additional file 1 **A list of 97 genes was differentially expressed which were previously reported to be involved in cancer**. Click here for file

Additional file 2 **The list of 1697 differentially expressed genes**. Click here for file

Additional file 3 **40 differentially expressed genes with damage values greater than 15**. Click here for file

Additional file 4 **A total of 149 differentially expressed miRNA**. Click here for file

Additional file 5 **Top 19 differentially expressed miRNAs with the largest damage values (> 20) in the miRNA coexpression network**. Click here for file

Additional file 6 **3,953 matched miRNA-mRNA pairs**. Click here for file

Additional file 7 **A total of 34 differentially expressed miRNA negatively regulates 17 genes with damage values greater than 19**. Click here for file

Additional file 8 **Target genes of top 19 miRNAs selected according to damage values of miRNA**. Click here for file

Additional file 9 **A total of 78 target genes with coefficient of determination greater than 20%**. Click here for file

Additional file 10 **177 significantly differentially expressed genes regulated by somatic mutation in 14 genes**. Click here for file

Additional file 11 **A total of 23 miRNAs were regulated by somatic mutations**. Click here for file

Additional file 12 **A total of 323 differentially expressed genes were regulated by LOH in the 11 genes associated with GBM**. Click here for file

Additional file 13 **A total of 19 miRNAs were regulated by LOH mutations**. Click here for file

Additional file 14 **Enriched GO and pathways targeted by Somatic and LOH mutation eQTL**. Click here for file

## References

[B1] Lipsitz D Higgins RJ Kortz GD Dickinson PJ Bollen AW Naydan DK LeCouteur RA Glioblastoma multiforme: clinical findings, magnetic resonance imaging, and pathology in five dogs Vet Pathol 2003 40 659 669 10.1354/vp.40-6-659 14608019

[B2] Mischel PS Nelson SF Cloughesy TF Molecular analysis of glioblastoma: pathway profiling and its implications for patient therapy Cancer Biol Ther 2003 2 242 247 12878856 10.4161/cbt.2.3.369

[B3] Krex D Klink B Hartmann C von Deimling A Pietsch T Simon M Sabel M Steinbach JP Heese O Reifenberger G Long-term survival with glioblastoma multiforme Brain 2007 130 2596 2606 10.1093/brain/awm204 17785346

[B4] The Cancer Genome Atlas Research Network Comprehensive genomic characterization defines human glioblastoma genes and core pathways Nature 2008 455 1061 1068 10.1038/nature07385 18772890 PMC2671642

[B5] Gennarino VA Sardiello M Avellino R Meola N Maselli V Anand S Cutillo L Ballabio A Banfi S MicroRNA target prediction by expression analysis of host genes Genome Res 2009 19 481 490 10.1101/gr.084129.108 19088304 PMC2661810

[B6] Maziere P Enright AJ Prediction of microRNA targets Drug Discov Today 2007 12 452 458 10.1016/j.drudis.2007.04.002 17532529

[B7] Yang Y Wang YP Li KB MiRTif: a support vector machine-based microRNA target interaction filter BMC Bioinformatics 2008 9 Suppl 12 S4 10.1186/1471-2105-9-S12-S4 19091027 PMC2638144

[B8] Michaelson JJ Loguercio S Beyer A Detection and interpretation of expression quantitative trait loci (eQTL) Methods 2009 48 265 276 10.1016/j.ymeth.2009.03.004 19303049

[B9] Dartnell L Simeonidis E Hubank M Tsoka S Bogle ID Papageorgiou LG Robustness of the p53 network and biological hackers FEBS Lett 2005 579 3037 3042 10.1016/j.febslet.2005.03.101 15896791

[B10] Demetrius L Manke T Robustness and network evolution-an entropic principle Physica A: Statistical Mechanics and its Applications 2005 346 682 10.1016/j.physa.2004.07.011

[B11] Azzopardi D Dallosso AR Eliason K Hendrickson BC Jones N Rawstorne E Colley J Moskvina V Frye C Sampson JR Multiple rare nonsynonymous variants in the adenomatous polyposis coli gene predispose to colorectal adenomas Cancer Res 2008 68 358 363 10.1158/0008-5472.CAN-07-5733 18199528

[B12] Madsen BE Browning SR A groupwise association test for rare mutations using a weighted sum statistic PLoS Genet 2009 5 e1000384 10.1371/journal.pgen.1000384 19214210 PMC2633048

[B13] Xiong M Zhao J Boerwinkle E Generalized T2 test for genome association studies Am J Hum Genet 2002 70 1257 1268 10.1086/340392 11923914 PMC447600

[B14] Li B Leal SM Methods for detecting associations with rare variants for common diseases: application to analysis of sequence data Am J Hum Genet 2008 83 311 321 10.1016/j.ajhg.2008.06.024 18691683 PMC2842185

[B15] Joyce P Tavare S The distribution of rare alleles J Math Biol 1995 33 602 618 10.1007/BF00298645 7608640

[B16] Maglott D Ostell J Pruitt KD Tatusova T Entrez Gene: gene-centered information at NCBI Nucleic Acids Res 2007 35 D26 31 10.1093/nar/gkl993 17148475 PMC1761442

[B17] Liang P Pardee AB Analysing differential gene expression in cancer Nat Rev Cancer 2003 3 869 876 10.1038/nrc1214 14668817

[B18] Knorn F Ranking schemes. In Ranking and importance in complex networks 2005 Kildare Ireland: National University of Ireland Maynooth, Co

[B19] El Hallani S Ducray F Idbaih A Marie Y Boisselier B Colin C Laigle-Donadey F Rodero M Chinot O Thillet J TP53 codon 72 polymorphism is associated with age at onset of glioblastoma Neurology 2009 72 332 336 10.1212/01.wnl.0000341277.74885.ec 19171829

[B20] Imoto I Pimkhaokham A Watanabe T Saito-Ohara F Soeda E Inazawa J Amplification and overexpression of TGIF2, a novel homeobox gene of the TALE superclass, in ovarian cancer cell lines Biochem Biophys Res Commun 2000 276 264 270 10.1006/bbrc.2000.3449 11006116

[B21] Yang HS Cho MH Zakowicz H Hegamyer G Sonenberg N Colburn NH A novel function of the MA-3 domains in transformation and translation suppressor Pdcd4 is essential for its binding to eukaryotic translation initiation factor 4A Mol Cell Biol 2004 24 3894 3906 10.1128/MCB.24.9.3894-3906.2004 15082783 PMC387765

[B22] Turashvili G Bouchal J Baumforth K Wei W Dziechciarkova M Ehrmann J Klein J Fridman E Skarda J Srovnal J Novel markers for differentiation of lobular and ductal invasive breast carcinomas by laser microdissection and microarray analysis BMC Cancer 2007 7 55 10.1186/1471-2407-7-55 17389037 PMC1852112

[B23] Helleman J Jansen MP Span PN van Staveren IL Massuger LF Meijer-van Gelder ME Sweep FC Ewing PC van der Burg ME Stoter G Molecular profiling of platinum resistant ovarian cancer Int J Cancer 2006 118 1963 1971 10.1002/ijc.21599 16287073

[B24] Sethi T Rintoul RC Moore SM MacKinnon AC Salter D Choo C Chilvers ER Dransfield I Donnelly SC Strieter R Haslett C Extracellular matrix proteins protect small cell lung cancer cells against apoptosis: a mechanism for small cell lung cancer growth and drug resistance in vivo Nat Med 1999 5 662 668 10.1038/9511 10371505

[B25] Cimino G Sprovieri T Rapanotti MC Foa R Mecucci C Mandelli F Molecular evaluation of the NUP98/RAP1GDS1 gene frequency in adults with T-acute lymphoblastic leukemia Haematologica 2001 86 436 437 11325654

[B26] Hussey DJ Nicola M Moore S Peters GB Dobrovic A The (4;11)(q21;p15) translocation fuses the NUP98 and RAP1GDS1 genes and is recurrent in T-cell acute lymphocytic leukemia Blood 1999 94 2072 2079 10477737

[B27] Forch P Valcarcel J Molecular mechanisms of gene expression regulation by the apoptosis-promoting protein TIA-1 Apoptosis 2001 6 463 468 10.1023/A:1012441824719 11595836

[B28] Brooks AS Bertoli-Avella AM Burzynski GM Breedveld GJ Osinga J Boven LG Hurst JA Mancini GM Lequin MH de Coo RF Homozygous nonsense mutations in KIAA1279 are associated with malformations of the central and enteric nervous systems Am J Hum Genet 2005 77 120 126 10.1086/431244 15883926 PMC1226183

[B29] Sun S Ning X Liu J Liu L Chen Y Han S Zhang Y Liang J Wu K Fan D Overexpressed CacyBP/SIP leads to the suppression of growth in renal cell carcinoma Biochem Biophys Res Commun 2007 356 864 871 10.1016/j.bbrc.2007.03.080 17400182

[B30] Ning X Sun S Hong L Liang J Liu L Han S Liu Z Shi Y Li Y Gong W Calcyclin-binding protein inhibits proliferation, tumorigenicity, and invasion of gastric cancer Mol Cancer Res 2007 5 1254 1262 10.1158/1541-7786.MCR-06-0426 18171983

[B31] Huang da W Sherman BT Lempicki RA Systematic and integrative analysis of large gene lists using DAVID bioinformatics resources Nat Protoc 2009 4 44 57 10.1038/nprot.2008.211 19131956

[B32] Huang JC Babak T Corson TW Chua G Khan S Gallie BL Hughes TR Blencowe BJ Frey BJ Morris QD Using expression profiling data to identify human microRNA targets Nat Methods 2007 4 1045 1049 10.1038/nmeth1130 18026111

[B33] Zhang L Volinia S Bonome T Calin GA Greshock J Yang N Liu CG Giannakakis A Alexiou P Hasegawa K Genomic and epigenetic alterations deregulate microRNA expression in human epithelial ovarian cancer Proc Natl Acad Sci USA 2008 105 7004 7009 10.1073/pnas.0801615105 18458333 PMC2383982

[B34] Jiang Q Wang Y Hao Y Juan L Teng M Zhang X Li M Wang G Liu Y miR2Disease: a manually curated database for microRNA deregulation in human disease Nucleic Acids Res 2009 37 D98 104 10.1093/nar/gkn714 18927107 PMC2686559

[B35] Volinia S Galasso M Costinean S Tagliavini L Gamberoni G Drusco A Marchesini J Mascellani N Sana ME Abu Jarour R Reprogramming of miRNA networks in cancer and leukemia Genome Res 2010 20 589 599 10.1101/gr.098046.109 20439436 PMC2860161

[B36] Guo AY Sun J Jia P Zhao Z A novel microRNA and transcription factor mediated regulatory network in schizophrenia BMC Syst Biol 2010 4 10 10.1186/1752-0509-4-10 20156358 PMC2834616

[B37] Song F Zheng H Liu B Wei S Dai H Zhang L Calin GA Hao X Wei Q Zhang W Chen K An miR-502-binding site single-nucleotide polymorphism in the 3'-untranslated region of the SET8 gene is associated with early age of breast cancer onset Clin Cancer Res 2009 15 6292 6300 10.1158/1078-0432.CCR-09-0826 19789321

[B38] Cox DR Regression models and life-tables J Roy Statist Soc SerB Methodological 1972 34 24

[B39] Sassen S Miska EA Caldas C MicroRNA: implications for cancer Virchows Arch 2008 452 1 10 10.1007/s00428-007-0532-2 18040713 PMC2151131

[B40] Megraw M Sethupathy P Corda B Hatzigeorgiou AG miRGen: a database for the study of animal microRNA genomic organization and function Nucleic Acids Res 2007 35 D149 155 10.1093/nar/gkl904 17108354 PMC1669779

[B41] Sethupathy P Corda B Hatzigeorgiou AG TarBase: A comprehensive database of experimentally supported animal microRNA targets RNA 2006 12 192 197 10.1261/rna.2239606 16373484 PMC1370898

[B42] Karginov FV Conaco C Xuan Z Schmidt BH Parker JS Mandel G Hannon GJ A biochemical approach to identifying microRNA targets Proc Natl Acad Sci USA 2007 104 19291 19296 10.1073/pnas.0709971104 18042700 PMC2148283

[B43] Selbach M Schwanhausser B Thierfelder N Fang Z Khanin R Rajewsky N Widespread changes in protein synthesis induced by microRNAs Nature 2008 455 58 63 10.1038/nature07228 18668040

[B44] Yang Y Chaerkady R Beer MA Mendell JT Pandey A Identification of miR-21 targets in breast cancer cells using a quantitative proteomic approach Proteomics 2009 9 1374 1384 10.1002/pmic.200800551 19253296 PMC3030979

[B45] Carpenter B Hill KJ Charalambous M Wagner KJ Lahiri D James DI Andersen JS Schumacher V Royer-Pokora B Mann M BASP1 is a transcriptional cosuppressor for the Wilms' tumor suppressor protein WT1 Mol Cell Biol 2004 24 537 549 10.1128/MCB.24.2.537-549.2004 14701728 PMC343806

[B46] Mingorance A Soriano-Garcia E del Rio JA [Nogo-A functions during the development of the central nervous system and in the adult] Rev Neurol 2004 39 440 446 15378458

[B47] Skalsky RL Samols MA Plaisance KB Boss IW Riva A Lopez MC Baker HV Renne R Kaposi's sarcoma-associated herpesvirus encodes an ortholog of miR-155 J Virol 2007 81 12836 12845 10.1128/JVI.01804-07 17881434 PMC2169101

[B48] Fasanaro P D'Alessandra Y Di Stefano V Melchionna R Romani S Pompilio G Capogrossi MC Martelli F MicroRNA-210 modulates endothelial cell response to hypoxia and inhibits the receptor tyrosine kinase ligand Ephrin-A3 J Biol Chem 2008 283 15878 15883 10.1074/jbc.M800731200 18417479 PMC3259646

[B49] Sengupta S den Boon JA Chen IH Newton MA Stanhope SA Cheng YJ Chen CJ Hildesheim A Sugden B Ahlquist P MicroRNA 29c is down-regulated in nasopharyngeal carcinomas, up-regulating mRNAs encoding extracellular matrix proteins Proc Natl Acad Sci USA 2008 105 5874 5878 10.1073/pnas.0801130105 18390668 PMC2311339

[B50] Li Z Hassan MQ Jafferji M Aqeilan RI Garzon R Croce CM van Wijnen AJ Stein JL Stein GS Lian JB Biological functions of miR-29b contribute to positive regulation of osteoblast differentiation J Biol Chem 2009 284 15676 15684 10.1074/jbc.M809787200 19342382 PMC2708864

[B51] Rayner KJ Suarez Y Davalos A Parathath S Fitzgerald ML Tamehiro N Fisher EA Moore KJ Fernandez-Hernando C MiR-33 contributes to the regulation of cholesterol homeostasis Science 2010 328 1570 1573 10.1126/science.1189862 20466885 PMC3114628

[B52] Herrera-Merchan A Cerrato C Luengo G Dominguez O Piris MA Serrano M Gonzalez S miR-33-mediated downregulation of p53 controls hematopoietic stem cell self-renewal Cell Cycle 2010 9 3277 3285 10.4161/cc.9.16.12598 20703086

[B53] Pacifico F Paolillo M Chiappetta G Crescenzi E Arena S Scaloni A Monaco M Vascotto C Tell G Formisano S Leonardi A RbAp48 is a target of nuclear factor-kappaB activity in thyroid cancer J Clin Endocrinol Metab 2007 92 1458 1466 10.1210/jc.2006-2199 17244783

[B54] Caligo MA Cipollini G Berti A Viacava P Collecchi P Bevilacqua G NM23 gene expression in human breast carcinomas: loss of correlation with cell proliferation in the advanced phase of tumor progression Int J Cancer 1997 74 102 111 10.1002/(SICI)1097-0215(19970220)74:1<102::AID-IJC18>3.0.CO;2-H 9036878

[B55] Hagedorn M Delugin M Abraldes I Allain N Belaud-Rotureau MA Turmo M Prigent C Loiseau H Bikfalvi A Javerzat S FBXW7/hCDC4 controls glioma cell proliferation in vitro and is a prognostic marker for survival in glioblastoma patients Cell Div 2007 2 9 10.1186/1747-1028-2-9 17326833 PMC1819378

[B56] Heraud C Chevrier L Meunier AC Muller JM Chadeneau C Vasoactive intestinal peptide-induced neuritogenesis in neuroblastoma SH-SY5Y cells involves SNAP-25 Neuropeptides 2008 42 611 621 10.1016/j.npep.2008.05.005 18617262

[B57] Wang C Li N Liu X Zheng Y Cao X A novel endogenous human CaMKII inhibitory protein suppresses tumor growth by inducing cell cycle arrest via p27 stabilization J Biol Chem 2008 283 11565 11574 10.1074/jbc.M800436200 18305109 PMC2431061

[B58] Hansen CN Ketabi Z Rosenstierne MW Palle C Boesen HC Norrild B Expression of CPEB, GAPDH and U6snRNA in cervical and ovarian tissue during cancer development APMIS 2009 117 53 59 10.1111/j.1600-0463.2008.00015.x 19161537

[B59] Namkoong J Shin SS Lee HJ Marin YE Wall BA Goydos JS Chen S Metabotropic glutamate receptor 1 and glutamate signaling in human melanoma Cancer Res 2007 67 2298 2305 10.1158/0008-5472.CAN-06-3665 17332361

[B60] Anglesio MS Arnold JM George J Tinker AV Tothill R Waddell N Simms L Locandro B Fereday S Traficante N Mutation of ERBB2 provides a novel alternative mechanism for the ubiquitous activation of RAS-MAPK in ovarian serous low malignant potential tumors Mol Cancer Res 2008 6 1678 1690 10.1158/1541-7786.MCR-08-0193 19010816 PMC6953412

[B61] Greenman C Stephens P Smith R Dalgliesh GL Hunter C Bignell G Davies H Teague J Butler A Stevens C Patterns of somatic mutation in human cancer genomes Nature 2007 446 153 158 10.1038/nature05610 17344846 PMC2712719

[B62] Wood LD Parsons DW Jones S Lin J Sjoblom T Leary RJ Shen D Boca SM Barber T Ptak J The genomic landscapes of human breast and colorectal cancers Science 2007 318 1108 1113 10.1126/science.1145720 17932254

[B63] Parsons DW Jones S Zhang X Lin JC Leary RJ Angenendt P Mankoo P Carter H Siu IM Gallia GL An integrated genomic analysis of human glioblastoma multiforme Science 2008 321 1807 1812 10.1126/science.1164382 18772396 PMC2820389

[B64] Rubin AF Green P Comment on "The consensus coding sequences of human breast and colorectal cancers" Science 2007 317 1500 10.1126/science.1138956 17872429

[B65] Yu SL Chen HY Chang GC Chen CY Chen HW Singh S Cheng CL Yu CJ Lee YC Chen HS MicroRNA signature predicts survival and relapse in lung cancer Cancer Cell 2008 13 48 57 10.1016/j.ccr.2007.12.008 18167339

[B66] Peng J Wang P Zhou NF Zhu J Partial correlation estimation by joint sparse regression model Journal of the American Statistical Association 2009 104 735 10.1198/jasa.2009.0126 19881892 PMC2770199

[B67] Langfelder P Zhang B Horvath S Defining clusters from a hierarchical cluster tree: the Dynamic Tree Cut package for R Bioinformatics 2008 24 719 720 10.1093/bioinformatics/btm563 18024473

[B68] Hashimoto K Goto S Kawano S Aoki-Kinoshita KF Ueda N Hamajima M Kawasaki T Kanehisa M KEGG as a glycome informatics resource Glycobiology 2006 16 63R 70R 10.1093/glycob/cwj010 16014746

